# Tentacles Can Reach the Duodenum: A Rare Form of Gastritis

**DOI:** 10.7759/cureus.68288

**Published:** 2024-08-31

**Authors:** Magdalini Manti, Alexandros Toskas, Brian Saunders

**Affiliations:** 1 Gastroenterology, St Mark's Hospital, London, GBR

**Keywords:** anaemia, duodenum, hypoalbuminaemia, helicobacter pylori, varioliform gastritis

## Abstract

Varioliform gastritis (VG) is a rare chronic gastritis characterized by mucosal protrusions with central depressions, typically found in the stomach. This paper discusses the first reported case of VG extending into the duodenum, involving a 68-year-old immunocompromised patient with a complex medical history, including prostate cancer and multiple comorbidities. The diagnosis was complicated by the presence of *Helicobacter pylori*, which was treated successfully with eradication therapy consisting of amoxicillin and clarithromycin along with omeprazole. Highlighting the potential for VG to affect areas beyond the stomach, this case underscores the importance of considering VG in patients with unexplained hypoalbuminemia and gastrointestinal symptoms.

## Introduction

Varioliform gastritis (VG), first described by Moutier and Martin in 1947, is a rare form of chronic gastritis, characterized endoscopically by multiple mucosal protrusions, often with a central depression or erosion [1]. Also known as octopus sucker gastritis, it is commonly associated with hyperplasia of the fundic folds [2]. The incidence of VG ranges from 0.5% to 2.8% of all gastritis cases, with a higher prevalence observed in male patients [1].

VG is classified into two distinct types based on its pathogenesis: diffuse, which is linked to allergic mechanisms, and antral, which is associated with ulcers, liver cirrhosis, and cholelithiasis [2]. The pathogenesis of VG remains an area of ongoing research, with recent studies suggesting that *Helicobacter pylori* (*H. pylori*) may serve as a significant contributing factor, particularly in the antral form. *H. pylori* is well-known for its role in various gastric pathologies, including peptic ulcer disease and gastric carcinoma, and its potential involvement in VG adds another layer of complexity to this condition [3].

The histological features of VG include mucosal infiltration by chronic inflammatory cells, often accompanied by oedema and hyperplasia of the gastric epithelium. These changes can lead to the characteristic nodularity seen in VG, which is distinct from the more common presentations of chronic gastritis. In some cases, VG may coexist with other forms of gastritis, such as lymphocytic or eosinophilic gastritis, further complicating the clinical picture. Lymphocytic gastritis (LG), identified in 1986, represents another rare subtype of chronic gastritis, comprising about 1.5% of gastritis biopsies. Also known as verrucous gastritis or octopus sucker gastritis, this condition is characterized by the accumulation of intraepithelial lymphocytes within the gastric mucosa [2]. This condition has been linked to a variety of underlying diseases, including celiac disease, *H. pylori* infection, Crohn's disease, Menetrier's disease, autoimmune disorders, and lymphomas.

The clinical significance of VG extends beyond its rarity; it has been associated with serious complications, such as hypoalbuminaemia, which can lead to oedema and other systemic effects. The exact mechanisms by which VG contributes to these complications are not fully understood, but it is hypothesized that the chronic inflammatory state and mucosal damage characteristic of VG may play a central role [3].

In this report, we present a unique case of VG with duodenal involvement, attributed to *H. pylori* infection, in an immunocompromised patient presenting with melena. This case contributes to the growing understanding of VG's potential to affect areas beyond the stomach and underscores the importance of considering VG in patients with atypical gastrointestinal presentations.

## Case presentation

**Figure 1:**
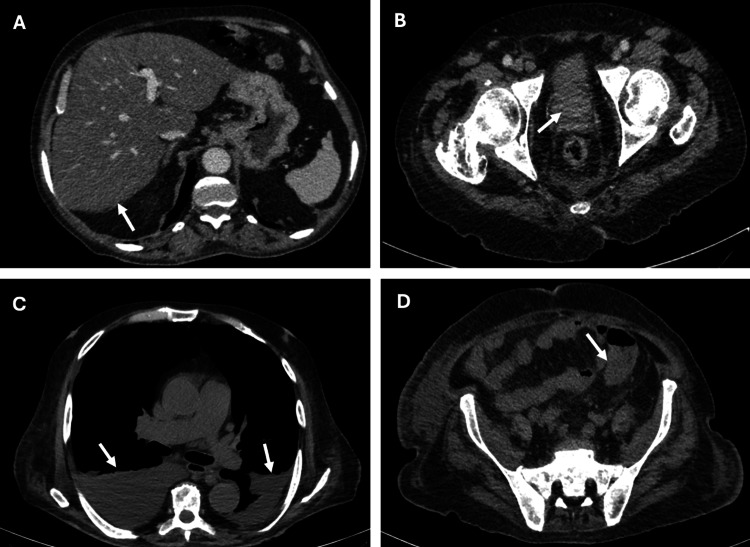
CT scans A) Previous CT abdomen: diffuse liver steatosis (arrow). B) Previous CT-abdomen: prostate malignancy (arrow). C) Current CT angiogram: moderate-sized bilateral pleural effusions, larger on the right (arrows). D) Current CT angiogram: fluid-filled rectosigmoid (arrow) with no convincing evidence of contrast blush to suggest active haemorrhage. There is some apparent wall irregularity of the distal sigmoid and the rectum.

A 68-year-old man was admitted with confusion, drowsiness, and bilateral peripheral leg oedema. He was housebound, living with caregivers, and had a performance status of three. He was recently diagnosed with T3N1M1b prostate cancer with vertebral bone metastases, and he was deemed fit only for hormonal treatment. His medical history included treated hypertension, type 2 diabetes and liver steatosis, as shown in a previous CT scan (Figure 1A). Although a non-smoker, he had been consuming 30 units of alcohol daily for the past five months. His regular medications included bicalutamide, gliclazide, amlodipine, ramipril, cholecalciferol, folic acid, thiamine, and tadalafil.

Upon admission, blood tests revealed hypoglycemia, macrocytic anaemia, and hypoalbuminaemia. His ECG showed newly diagnosed atrial fibrillation with a fast ventricular response, while the echocardiogram was unremarkable, showing normal ejection fraction, no regional wall motion abnormalities, and no valvular disease (Table 1). The patient was subsequently started on a therapeutic dose of oral anticoagulation (apixaban).

**Table 1:** Echocardiography report LV - left ventricle, LVEF - left ventricular ejection fraction, LA - left atrium, RA - right atrium, RV - right ventricle, IVC - inferior vena cava

Cardiac parameters	Findings
Rhythm and rate	Atrial fibrillation, 78bpm
Left ventricle	Normal LV dimensions and wall thickness with overall borderline low LV systolic function. No obvious resting regional wall motion abnormalities. Biplane LVEF 50%
Right ventricle	Normal size and normal systolic function
Atria	LA and RA of normal size
Heart valves	All valves appear structurally normal with trivial tricuspid regurgitation
IVC	normal size with good respiratory variation

A few days later, he developed bloody diarrhoea, leading to a drop in his haemoglobin level from 107 g/L to 83 g/L (Table 2). Anticoagulation was promptly discontinued, and he was urgently evaluated with a CT angiogram (Figure 1) and sigmoidoscopy.

**Table 2:** Laboratory results HB - haemoglobulin, MCV - mean corpuscular volume, pro-BNP - pro-brain natriuretic peptide

investigation	Results	Normal range
Hb	107g/L (on admission) 83g/L (during haematochezia) 76g/L (during melaena)	130-170 g/L
MCV	104.6 fL	80-100 fL
Glucose	3.8 mmol/L	4-7mmol/L
Ferritin	1706 ng/mL	30-400 ng/mL
Folate	>20 ng/mL	3-17 ng/mL
Serum albumin	2.2 g/dL	3.5-5.0 g/dL
Pro-BNP	1616 pg/mL	<125 pg/mL

CT showed bilateral pleural effusions and mild wall irregularity of the distal sigmoid and the rectum without evidence of active bleeding. His flexible sigmoidoscopy demonstrated patchy erythema with central ulceration, but histology did not confirm any signs of ischaemia.

The stool culture tested negative for toxins but was positive for *Clostridium difficile* antigen, prompting treatment with oral vancomycin. After five days, his rectal bleeding and diarrhoea resolved, allowing for the reintroduction of anticoagulation therapy.

**Figure 2:**
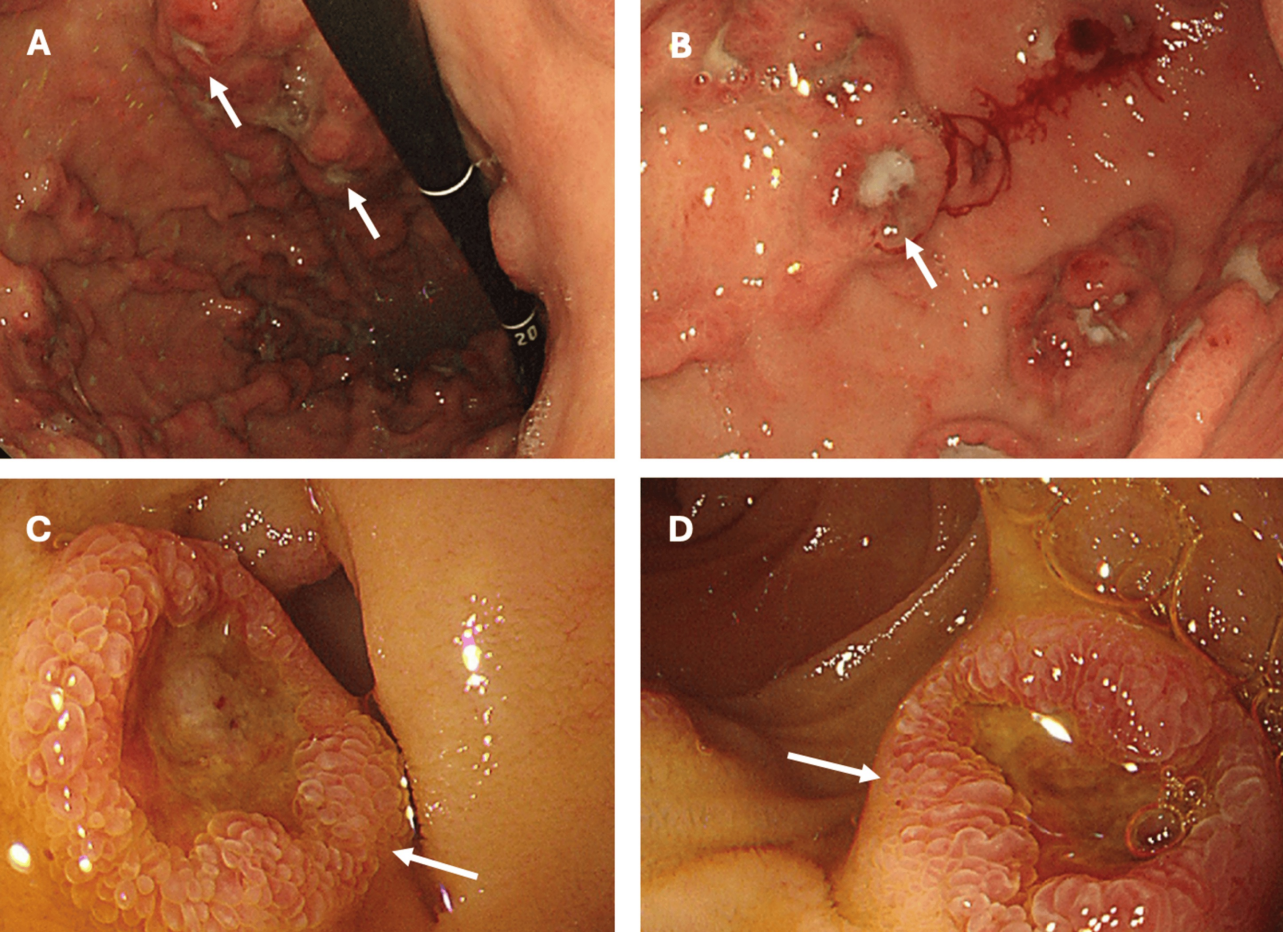
Endoscopic images Nodular lesions with central star-shaped ulceration (arrows) on the fundus (A), antrum (B), first (C), and second part of the duodenum (D)

On day 27 of his admission, the patient experienced further episodes of melaena, accompanied by a drop in haemoglobin to 76 g/L. A subsequent gastroscopy revealed more than 10 nodular lesions with central, star-shaped ulcerations located in the fundus, gastric corpus, and antrum. Additionally, up to 10 similar lesions were unexpectedly found in the first and second parts of the duodenum (Figure 2). Gastric biopsies confirmed *Helicobacter pylori-*positive gastritis with florid regenerative changes, while duodenal biopsies indicated focal ulceration, granulation tissue, intraepithelial lymphocytes, and regenerative changes consistent with varioliform changes.

In response to the *H. pylori* diagnosis, the patient was started on empirical eradication therapy, including amoxicillin, clarithromycin, and omeprazole. He responded well to the treatment and was discharged 10 days later, with a follow-up endoscopy scheduled for eight weeks post-discharge. Unfortunately, three weeks later, the patient was readmitted with hospital-acquired pneumonia and acute kidney injury, which led to anuria. Despite efforts, he was transitioned to palliative care and subsequently passed away in a hospice.

## Discussion

Varioliform gastritis is a relatively rare endoscopic finding, and this case represents the first reported instance of VG extending into the duodenum. This unusual presentation adds a new dimension to the understanding of VG's clinical spectrum. Notably, the patient had undergone a gastroscopy two months prior, which revealed an atypical spindle cell lesion but no evidence of VG or* H. pylori* infection. The histology from that earlier procedure, reviewed by a specialized histopathologist within the regional Sarcoma MDT, was characterized as granulation-type tissue with reactive fibroblastic proliferation, raising no initial suspicion of VG.

The patient's clinical course and the findings from the subsequent gastroscopy suggest that VG can progress rapidly, particularly in the presence of predisposing factors such as *H. pylori* infection and underlying liver disease. Observational studies have proposed that *H. pylori* may act as an initial trigger for antral VG, and this case supports that hypothesis. The patient's history of significant alcohol intake and diffuse liver steatosis, as seen on the CT scan, further aligns with findings from retrospective studies that have linked VG with cirrhosis and other liver conditions (p<0.01) [4]. Additionally, other factors such as atopic respiratory diseases, work-related stress, and irregular meals have been identified as potential contributors to VG (p<0.001) [5].

The patient's presentation with severe hypoalbuminaemia and oedema, both of which are recognized consequences of diffuse VG, underscores the multifactorial nature of his condition. While these symptoms could be attributed to various causes, VG likely did play a significant role. This is consistent with previous case studies that have reported hypoalbuminaemia as a consequence of VG [6].

Management of VG in this case involved *H. pylori* eradication therapy, which is recommended even in the absence of positive biopsy results. The patient's positive response to the eradication therapy, despite initially negative histology, suggests that empirical treatment should be strongly considered in similar cases. This approach is particularly important given the potential for VG to mimic other gastrointestinal conditions, complicating diagnosis.

Post-eradication, endoscopic, and histopathological surveillance remains critical, not only to assess remission [6] but also to exclude the possibility of malignancy - a significant concern in cases involving atypical gastric lesions. The involvement of a multidisciplinary team was instrumental in this case, particularly in ruling out malignancy, guiding treatment decisions, and managing the patient's complex clinical presentation.

## Conclusions

This case highlights a rare instance of varioliform gastritis extending into the duodenum, a condition previously undocumented in the literature. The patient's presentation with severe hypoalbuminaemia and multiple systemic complications underscores the need for clinicians to consider VG in differential diagnoses, especially in the presence of *H. pylori* infection and liver disease. The successful response to *H. pylori* eradication therapy, despite negative histology in prior evaluations, further supports the necessity of initiating treatment even in uncertain cases. This case also emphasises the importance of vigilant follow-up and the role of multidisciplinary teams in excluding malignancy and managing complex presentations. Ultimately, this report expands the understanding of VG's potential clinical manifestations and the critical importance of early intervention.

## References

[1] Cheli R, Perasso A, Giacosa A (1987). Varioliform gastritis. Gastritis.

[2] Kasper P, Loeser H, Goeser T (2018). Varioliform gastritis: an unusual endoscopic finding. Ann Gastroenterol.

[3] Pennelli G, Grillo F, Galuppini F (2020). Gastritis: update on etiological features and histological practical approach. Pathologica.

[4] Roblero JP, Iturriaga H, Estela R (2010). Association between varioliform gastritis and cirrhosis (Article in Spanish). Rev Med Chil.

[5] Zou TH, Zheng RH, Gao QY (2016). Factors affecting occurrence of gastric varioliform lesions: a case-control study. World J Gastroenterol.

[6] Zimmer V, Emrich K (2022). Varioliform-type lymphocytic gastritis: treating H. pylori or (rather) something else?. Am J Med Sci.

